# Hemodialysis and erythrocyte epoxy fatty acids

**DOI:** 10.14814/phy2.14601

**Published:** 2020-10-28

**Authors:** Benjamin Gollasch, Guanlin Wu, Tong Liu, Inci Dogan, Michael Rothe, Maik Gollasch, Friedrich C. Luft

**Affiliations:** ^1^ Experimental and Clinical Research Center (ECRC) A Joint Institution Between the Charité University Medicine and Max Delbrück Center (MDC) for Molecular Medicine Berlin‐Buch Germany; ^2^ HELIOS Klinikum Berlin‐Buch Berlin Germany; ^3^ Max Delbrück Center for Molecular Medicine (MDC) in the Helmholtz Association Berlin Germany; ^4^ LIPIDOMIX GmbH Berlin Germany; ^5^ Nephrology/Intensive Care Section Charité Campus Virchow Berlin Germany; ^6^ Department of Internal and Geriatric Medicine University Medicine Greifswald Greifswald Germany

**Keywords:** chronic kidney disease (CKD), dialysis, erythrocytes, fatty acids, lipidomics

## Abstract

Fatty acid products derived from cytochromes P450 (CYP) monooxygenase and lipoxygenase (LOX)/CYP ω/(ω‐1)‐hydroxylase pathways are a superclass of lipid mediators with potent bioactivities. Whether or not the chronic kidney disease (CKD) and hemodialysis treatments performed on end‐stage renal disease (ESRD) patients affect RBC epoxy fatty acids profiles remains unknown. Measuring the products solely in plasma is suboptimal. Since such determinations invariably ignore red blood cells (RBCs) that make up 3 kg of the circulating blood. RBCs are potential reservoirs for epoxy fatty acids that regulate cardiovascular function. We studied 15 healthy persons and 15 ESRD patients undergoing regular hemodialysis treatments. We measured epoxides derived from CYP monooxygenase and metabolites derived from LOX/CYP ω/(ω‐1)‐hydroxylase pathways in RBCs by LC–MS/MS tandem mass spectrometry. Our data demonstrate that various CYP epoxides and LOX/CYP ω/(ω‐1)‐hydroxylase products are increased in RBCs of ESRD patients, compared to control subjects, including dihydroxyeicosatrienoic acids (DHETs), epoxyeicosatetraenoic acids (EEQs), dihydroxydocosapentaenoic acids (DiHDPAs), and hydroxyeicosatetraenoic acids (HETEs). Hemodialysis treatment did not affect the majority of those metabolites. Nevertheless, we detected more pronounced changes in free metabolite levels in RBCs after dialysis, as compared with the total RBC compartment. These findings indicate that free RBC eicosanoids should be considered more dynamic or vulnerable in CKD.

## Graphical abstract

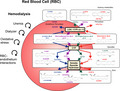

**Abbreviations**
AA: arachidonic acid, C20:4
CYP: cytochrome P450
DHA: docosahexaenoic acid, C22:6 n‐3
DHET: dihydroxyeicosatrienoic acid
DiHDHA: dihydroxydocosahexaenoic acid
DiHDPA: dihydroxydocosapentaenoic acid
DiHETE: dihydroxyeicosatetraenoic acid
DiHOME: dihydroxyctadecenoic acid
EDHF: endothelium‐derived hyperpolarizing factor
EDP: epoxydocosapentaenoic acid
EEQ: epoxyeicosatetraenoic acid
EET: epoxyeicosatrienoic acid
EPA: eicosapentaenoic acid, C20:5 n‐3
EpOME: epoxyoctadecenoic acid
HDHA: hydroxydocosahexaenoic acid
HEPE: hydroxyeicosapentaenoic acid
HETE: hydroxyeicosatetraenoic acid
HODE: hydroxyoctadecadienoic acid
HPETE: hydroperoxyeicosatetraenoic acid
HpODE: hydroperoxylinoleic acid
LA: linoleic acid, C18:2
LOX: lipoxygenase
PUFA: polyunsaturated fatty acid

## 1 INTRODUCTION

Chronic kidney disease (CKD) is a risk factor for the composite outcome of all‐cause mortality and cardiovascular disease (Weiner et al., 2004). Although mortality and cardiovascular disease burden have decreased for end‐stage renal disease (ESRD) hemodialysis patients in the United States, the 5‐year mortality is still ~50% (McGill et al., 2019), Most of these deaths are related to cardiovascular disease (CVD) (Felasa | Federation for Laboratory Animal Science Associations, 2012; Luft, 2000). Dietary omega‐3 (n‐3) fatty acid intake is associated with a reduced CVD risk (Harris et al., 2008; Huang et al., 2011; InterAct Consortium et al., 2011). Erythrocyte red‐blood‐cell (RBC) n‐3 fatty‐acid status is inversely related to cardiovascular events, such as cardiac arrhythmias, myocardial infarction, and sudden cardiac death (Bucher et al., 2002).

**Figure 1:**
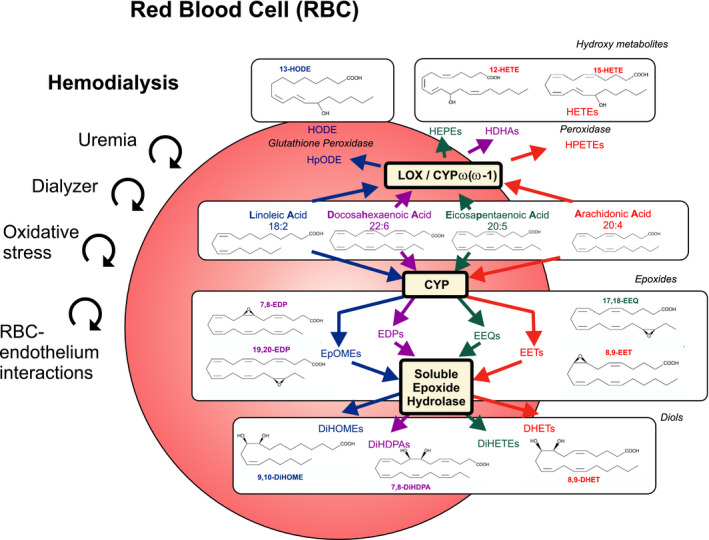
Hypothetic influence of CKD and hemodialysis associated with shear stress, red blood cell (RBC)‐dialyzer interactions, red blood cell (RBC)‐endothelial interactions, and oxidative stress affecting the content of cytochrome P450 epoxygenase (CYP) and 12‐ and 15‐lipoxygenase (LOX)/CYP omega‐hydroxylase metabolites in RBCs. The scheme illustrates the epoxide and hydroxy metabolites pathways studied. Linoleic (LA), arachidonic (AA), eicosapentaenoic (EPA), and docosahexaenoic acids (DHA) are converted to epoxyoctadecenoic acids (EpOMEs, e.g., 9,10‐EpOME), epoxyeicosatrienoic acid (EETs, e.g., 8,9‐EET), epoxyeicosatetraenoic acids (EEQs, e.g., 17,18‐EEQ), and epoxydocosapentaenoic acids (EDPs, e.g., 17,18‐EDP and 19,20‐EDP) by CYP, respectively. EpOMEs, EETs, EEQs, and EDPs are converted to dihydroxyctadecenoic acids (DiHOMEs, e.g., 9,10‐DiHOME), dihydroxyeicosatrienoic acids (DHETs, e.g., 8,9‐DHET), dihydroxyeicosatetraenoic acids (DiHETEs), and dihydroxydocosapentaenoic acids (DiHDPAs, e.g., 7,8‐DiHDPA), respectively, by the soluble epoxide hydrolase (sEH) enzyme. LA, AA, EPA, and DHA are converted to hydroperoxylinoleic acids (HpODEs), hydroxyoctadecadienoic acids (HODEs, e.g., 13‐HODE), hydroxydocosahexaenoic acids (HDHAs), hydroperoxyeicosatetraenoic acids (HPETEs), and hydroxyeicosatetraenoic acids (HETEs, e.g., 12‐HETE and 15‐HETE) by LOX, CYP omega/(omega‐1)‐hydroxylase and peroxidase pathways. The metabolites measured within these pathways track the changes observed. Arrows demarcate metabolic pathways evaluated

Epoxides and hydro(pero)xy fatty acids (or oxylipins) are lipid peroxidation products of polyunsaturated fatty acids (PUFA), including C18:2 linoleic (LA), C20:0 arachidonic (AA), C20:5 n‐3 eicosapentaenoic (EPA), and C22:5 n‐3 docosahexaenoic acids (DHA). These products are derived from CYP monooxygenase, cyclooxygenase (COX), and LOX/CYP ω/(ω‐1)‐hydroxylase pathways, which catalyze the production in a highly tissue‐dependent and regioisomer‐specific manner (Figure 1). The resulting products are epoxyoctadecenoic acids (EpOMEs), epoxyeicosatrienoic acid (EETs), epoxyeicosatetraenoic acids (EEQs), epoxydocosapentaenoic acids (EDPs), hydroperoxylinoleic acids (HpODEs), hydroxyoctadecadienoic acids (HODEs), hydroxydocosahexaenoic acids (HDHAs), hydroperoxyeicosatetraenoic acids (HPETEs), and hydroxyeicosatetraenoic acids (HETEs) (Figure 1). EpOMEs, EETs, EEQs, and EDPs are converted depending on cell type, into secondary eicosanoids and their metabolites. The major metabolic route of CYP epoxides is incorporation into phospholipids and hydrolysis to corresponding diols by the enzyme soluble epoxide hydrolase (sEH) (Spector & Kim, 2015). CYP‐derived EETs and other epoxides, such as 17,18‐EEQ, serve as endothelium‐derived hyperpolarizing factors (EDHFs) to cause vasodilation (Campbell et al., 1996; Hercule et al., 2007; Hu & Kim, 1993). Recently, RBCs (~3 kg in human body) have been identified as a reservoir for CYP epoxides, in particular EETs, which on release may act in a vasoregulatory capacity (Jiang et al., 2010, 2011). Maximal exercise has been found to increase such erythro‐epoxides in RBCs, including 9,10‐EpOME, 12,13‐EpOME, 5,6‐EET, 11,12‐EET, 14,15‐EET, 16,17‐EDP, and 19,20‐EDP (Gollasch et al., 2019). Furthermore, sEH in the RBC and the resulting increase in EETs presumably contribute to a greater degree on regional blood flow than sEH inhibition localized in the arterial wall (Jiang et al., 2011; Yu et al., 2004). Nonetheless, the impact of epoxy and hydroxy fatty acids measurements in the RBCs for the prediction of CVD and mortality have not been previously elucidated. Whether or not CKD or hemodialysis treatment itself affect RBC‐epoxids and hydroxy metabolites remains unknown. We tested the hypotheses that CKD and hemodialysis treatments performed on end‐stage renal disease (ESRD) patients affect RBC epoxy fatty acids profiles.

## 2 METHODS

The Charité University Medicine Institutional Review Board approved this duly registered study (ClinicalTrials.gov, Identifier: NCT03857984). Recruitment was primarily *via* person‐to‐person interview. Prior to participation in the study, 15 healthy volunteers (6 male and 9 female) and 15 CKD patients (7 male and 8 female) undergoing regular hemodialysis treatment signed informed consent forms which outlined the treatments to be taken and the possible risks involved. All healthy control subjects were not taking medications. Venous blood was collected in each healthy subject by subcutaneous arm vein puncture in the sitting position. In the group of dialyzed patients (CKD group), all the blood samples were collected on the fistula arm right before beginning of the dialysis (starting of the HD, pre‐HD) and at the end of the dialysis (5–15 min before termination, post‐HD). Patients underwent thrice‐weekly dialysis, which lasted from 3 hr 45 min to 5 hr, based on high flux AK 200 dialyzers (Gambro GmbH, Hechingen, Germany). All samples were analyzed for RBC lipids. All blood samples were obtained by 4°C precooled EDTA vacuum extraction tube systems. Cells were separated from plasma by centrifugation for 10 min at 1,000–2,000 *g* using a refrigerated centrifuge RBCs were separated from EDTA blood by centrifugation as previously described (Gollasch, et al., 2020). RBC lipidomics was performed using LC–MS/MS tandem mass spectrometry as described in (Fischer et al., 2014; Gollasch et al., 2019; Gollasch et al., 2019). Concentrations are given in nanogram/g.

Descriptive statistics were calculated and variables were examined for meeting assumptions of normal distribution without skewness and kurtosis. In order to determine statistical significance, *t* test or Mann–Whitney test was used to compare the values of CKD versus control groups. Paired *t*‐test or paired Wilcoxon test were used to compare pre‐HD versus post‐HD values. In order to determine statistical significance between the four classes of epoxy‐metabolites hydrolyzed to appear in the circulation, Friedman's test followed by applying Dunn's multiple comparison test was used. In order to determine statistical significance between the four classes of epoxy‐metabolites hydrolyzed to appear in the circulation, Friedman's test followed by applying Dunn's multiple comparison test was used. The analysis included Mauchly's test of sphericity followed by applying the test of within‐subjects effects with Greenhouse–Geisser correction to ensure sphericity assumption (Gollasch et al., 2019; Gollasch et al., 2019). The .05 level of significance (*p*) was chosen. All data are presented as mean ± *SD*. All statistical analyses were performed using SPSS Statistics software (IBM Corporation) or All‐Therapy statistics beta (AICBT Ltd).

## 3 RESULTS

### 3.1 Clinical characteristics

The age between ESRD patients and the healthy subjects was not different (50 ± 18 years vs. 47 ± 12 years, respectively, *p* > .05, *n* = 15 each). The body mass indices between the two groups were also not different (24.8 ± 3.4 kg/m^2^ and 24.7 ± 4.6 kg/m^2^, respectively, *p* > .05, *n* = 15 each). The patients in the group CKD were diagnosed for the following conditions: diabetes mellitus (*n* = 4 patients), hypertension (*n* = 3), membranous glomerulonephritis (*n* = 2), autosomal dominant polycystic kidney disease (*n* = 1), other or unknown (*n* = 5). Major cardiovascular complications in the CKD group included peripheral artery disease (*n* = 3), cardiovascular (*n* = 2) and cerebrovascular (*n* = 1) events. Subjects were Caucasians, with the exception of one Black patient in the CKD group and one Asian subject in the control group.

### 3.2 RBC epoxy and hydroxy metabolites in CKD

We first determined the total levels of various CYP epoxides and LOX/CYP ω/(ω‐1)‐hydroxylase products in RBCs of the HD patients (Table 1) and compared the results with the healthy control subjects. Total CYP epoxides were analyzed for each member (Table 1A) and together within the four subclasses (Table 2A). RBCs of hemodialysis patients showed increased total levels of various individual CYP epoxides, namely 8,9‐DHET, 14,15‐DHET, 5,6‐EEQ, 11,12‐EEQ, 14,15‐EEQ, 17,18‐EEQ, 7,8‐DiHDPA, 10,11‐DiHDPA, 13,14‐DiHDPA, and 16,17‐DiHDPA in the RBCs (Table 1A). EpOMEs, DiHOMEs, EETs, EDPs (with exception of 19,20‐EDP), and DiHETEs were not different between both groups (Table 1A). Free CYP epoxides in the RBCs were also not different or only slightly decreased (8,9‐EET, 14,15‐EET, and 5,6‐EEQ) in RBCs of hemodialysis patients. Nonetheless, our analysis of the four CYP epoxide classes demonstrates that ESRD patients can be discriminated from controls by characteristic increases in three epoxide classes, that is, signatures, namely increased levels of total DHETs, EEQs, and DiHDPAs in the RBCs, that is, 5,6‐DHET+8,9‐DHET+11,12‐DHET+14,15‐DHET, 5,6‐EEQ+8,9‐EEQ+11,12‐EEQ+14,15‐EEQ+17,18‐EEQ, and 7,8‐DiHDPA+10,11‐DiHDPA+13,14‐DiHDPA+16,17‐DiHDPA+19,20‐DiHDPA (Table 2A). We next inspected the total levels of various LOX/CYP ω/(ω‐1)‐hydroxylase products in RBCs of the HD patients (Table 1A). We found that 5‐HETE, 8‐HETE, 9‐HETE, 11‐HETE, 12‐HETE, 15‐HETE, and 19‐HETE levels were increased in the hemodialysis patients, whereas 13‐HODE, 16‐HETE, 17‐HETE, 18‐HETE, 20‐HETE, 12 ‐HpETE, 5‐HEPE, 8‐HEPE, 9‐HEPE, 12‐HEPE, 15‐HEPE, 18‐HEPE, 19‐HEPE, 20‐HEPE, 4‐HDHA, 7‐HDHA, 8‐HDHA, 10‐HDHA, 11‐HDHA, 13‐HDHA, 14‐HDHA, 16‐HDHA, 17‐HDHA, 20‐HDHA, 21‐HDHA, and 22‐HDHA levels, were normal or nondetectable (Table 1A). Of note, free LOX/CYP ω/(ω‐1)‐hydroxylase products were generally increased in RBCs of hemodialysis patients, with exception of 17‐HETE, 18‐HETE, 19‐HETE, 20‐HETE, 12‐HpETE, 19‐HEPE, 20‐HEPE, and 20‐HDHA which were normal or non‐detectable (Table 1B). Together, the findings indicate that ESRD patients show an altered RBC fatty acid metabolite status, that is, individual signature, which shows the accumulation of three CYP epoxide classes (DHETs, EEQs, and DiHDPAs) and various HETEs and other LOX/CYP ω/(ω‐1) metabolites in RBCs, the latter mostly accumulated in free state.

**Table 1:** Comparison of epoxy‐ and hydroxy‐metabolites between control subjects versus CKD patients before hemodialysis (HD) (*n* = 15 each)

Amount (ng/g)	Control (Mean ± *SD*)	HD (mean ± *SD*)	*p* value, *t* test (^#^Mann‐Whitney test)
(A) Total metabolites in RBCs			
CYP epoxy‐metabolites			
(a) EpOMEs/DiHOMES			
9,10‐EpOME	29.36 ± 12.01	25.48 ± 6.59	.267^#^
12,13‐EpOME	13.67 ± 9.22	10.62 ± 6.16	.305^#^
9,10‐DiHOME	4.12 ± 1.30	5.13 ± 1.92	.081^#^
12,13‐DiHOME	2.26 ± 0.90	2.92 ± 1.45	.161^#^
(b) EETs/DiHOMEs			
5,6‐EET	170.67 ± 29.90	148.54 ± 44.94	.124
8,9‐EET	39.03 ± 6.25	39.90 ± 9.00	.761
11,12‐EET	39.46 ± 5.51	37.86 ± 11.98	.644
14,15‐EET	66.17 ± 11.64	59.58 ± 22.69	.328
5,6‐DHET	0.89 ± 0.17	0.98 ± 0.43	.457
8,9‐DHET	**1.07 ± 0.23**	**2.03 ± 1.81**	**.001^#^**
11,12‐DHET	0.62 ± 0.14	0.96 ± 0.61	.081^#^
14,15‐DHET	**0.40 ± 0.05**	**0.51 ± 0.16**	**.030**
(c) EEQs/DiHETEs			
5,6‐EEQ	**41.54 ± 13.39**	**51.78 ± 98.53**	**.019^#^**
8,9‐EEQ	2.48 ± 0.89	3.51 ± 6.41	.126^#^
11,12‐EEQ	**2.09 ± 0.68**	**2.56 ± 4.74**	**.016^#^**
14,15‐EEQ	**1.44 ± 0.48**	**1.91 ± 3.57**	**.041^#^**
17,18‐EEQ	**3.25 ± 1.03**	**3.90 ± 7.31**	**.021^#^**
5,6‐DiHETE	0.21 ± 0.10	0.28 ± 0.49	.202^#^
8,9‐DiHETE	0.01 ± 0.01	0.01 ± 0.01	.776^#^
11,12‐DiHETE	0.01 ± 0.01	0.01 ± 0.01	.677^#^
14,15‐DiHETE	0.01 ± 0.01	0.01 ± 0.01	.697^#^
17,18‐DiHETE	0.01 ± 0.01	0.01 ± 0.01	.787^#^
(d) EDPs/DiHDPAs			
7,8‐EDP	15.58 ± 4.55	18.16 ± 12.19	.838^#^
10,11‐EDP	1.22 ± 0.43	1.35 ± 0.47	.463
13,14‐EDP	0.39 ± 0.25	0.44 ± 0.15	.158^#^
16,17‐EDP	4.49 ± 1.34	4.72 ± 1.78	.967^#^
19,20‐EDP	**6.72 ± 4.26**	**4.22 ± 1.52**	**.026^#^**
7,8‐DiHDPA	**0.21 ± 0.10**	**0.40 ± 0.30**	**.041^#^**
10,11‐DiHDPA	**0.50 ± 0.20**	**0.09 ± 0.05**	**.007**
13,14‐DiHDPA	**0.08 ± 0.02**	**0.11 ± 0.04**	**.037^#^**
16,17‐DiHDPA	**0.14 ± 0.03**	**0.19 ± 0.06**	**.022**
19,20‐DiHDPA	0.20 ± 0.07	0.26 ± 0.16	.187^#^
LOX/CYP ω/(ω−1) metabolites			
13‐HODE	69.46 ± 19.97	77.47 ± 18,89	.098^#^
5‐HETE	**38.43 ± 7.90**	**53.45 ± 14.83**	**.002**
8‐HETE	**27.30 ± 5.72**	**35.11 ± 10.20**	**.015**
9‐HETE	**27.49 ± 4.72**	**37.84 ± 9.77**	**.001**
11‐HETE	**41.90 ± 7.00**	**54.16 ± 14.84**	**.009**
12‐HETE	**32.71 ± 5.66**	**43.47 ± 12.68**	**.007**
15‐HETE	**74.29 ± 14.38**	**93.95 ± 24.59**	**.012**
16‐HETE	4.60 ± 0.82	4.91 ± 1.43	.461
17‐HETE	0.18 ± 0.03	0.22 ± 0.10	.512^#^
18‐HETE	0.24 ± 0.05	0.32 ± 0.21	.461^#^
19‐HETE	**0.26 ± 0.11**	**0.42 ± 0.11**	**.001^#^**
20‐HETE	0.59 ± 0.09	0.62 ± 0.08	.371
12‐HpETE	n.d.	n.d.	n/a
5‐HEPE	1.47 ± 0.51	2.05 ± 2.64	.838^#^
8‐HEPE	0.75 ± 0.31	1.15 ± 1.55	.744^#^
9‐HEPE	0.93 ± 0.37	1.35 ± 1.64	.744^#^
12‐HEPE	1.38 ± 0.52	2.15 ± 3.12	.935^#^
15‐HEPE	1.18 ± 0.41	2.06 ± 2.74	.345^#^
18‐HEPE	3.19 ± 1.30	5.28 ± 7.10	.567^#^
19‐HEPE	1.32 ± 0.50	1.89 ± 2.80	.902^#^
20‐HEPE	n.d.	n.d.	n/a
4‐HDHA	9.11 ± 2.99	11.20 ± 4.61	.267^#^
7‐HDHA	4.56 ± 1.36	5.90 ± 2.69	.137^#^
8‐HDHA	5.27 ± 1.77	7.16 ± 3.11	.061^#^
10‐HDHA	6.39 ± 1.99	8.05 ± 3.79	.148
11‐HDHA	7.38 ± 2.41	9.43 ± 4.47	.217^#^
13‐HDHA	9.35 ± 2.80	10.43 ± 4.20	.414
14‐HDHA	5.41 ± 1.75	6.82 ± 3.38	.345^#^
16‐HDHA	8.79 ± 2.69	9.80 ± 3.88	.486^#^
17‐HDHA	12.98 ± 3.97	15.55 ± 6.92	.227
20‐HDHA	19.16 ± 5.89	22.57 ± 9.88	.261
21‐HDHA	3.04 ± 1.18	3.76 ± 1.70	.184
22‐HDHA	n.d.	n.d.	n/a
(B) Free metabolites in RBCs			
CYP epoxy‐metabolites			
(a) EpOMEs/DiHOMES			
9,10‐EpOME	1.42 ± 0.59	1.79 ± 1.00	.367^#^
12,13‐EpOME	1.22 ± 0.63	1.25 ± 0.91	.624^#^
9,10‐DiHOME	0.43 ± 0.29	0.52 ± 0.34	.595^#^
12,13‐DiHOME	1.70 ± 0.96	2.20 ± 1.52	.412^#^
(b) EETs/DiHOMEs			
5,6‐EET	0.55 ± 0.21	0.45 ± 0.19	.170
8,9‐EET	**0.12 ± 0.06**	**0.06 ± 0.04**	**.013^#^**
11,12‐EET	0.24 ± 0.07	0.20 ± 0.08	.100
14,15‐EET	**1.08 ± 0.40**	**0.74 ± 0.36**	**.015^#^**
5,6‐DHET	n.d.	n.d.	n/a
8,9‐DHET	n.d.	n.d.	n/a
11,12‐DHET	0.01 ± 0.01	0.01 ± 0.01	.467
14,15‐DHET	**0.01 ± 0.01**	**0.01 ± 0.01**	**.074^#^**
(c) EEQs/DiHETEs			
5,6‐EEQ	**1.29 ± 1.14**	**0.90 ± 3.39**	**.010^#^**
8,9‐EEQ	0.22 ± 0.12	0.31 ± 0.50	.351^#^
11,12‐EEQ	0.06 ± 0.04	0.07 ± 0.14	.116^#^
14,15‐EEQ	0.14 ± 0.10	0.20 ± 0.23	.851^#^
17,18‐EEQ	0.39 ± 0.19	0.53 ± 1.02	.217^#^
5,6‐DiHETE	n.d.	n.d.	n/a
8,9‐DiHETE	n.d.	n.d.	n/a
11,12‐DiHETE	n.d.	n.d.	n/a
14,15‐DiHETE	0.01 ± 0.01	0.01 ± 0.04	.285^#^
17,18‐DiHETE	0.04 ± 0.02	0.11 ± 0.23	.902^#^
(d) EDPs/DiHDPAs			
7,8‐EDP	0.12 ± 0.05	0.17 ± 0.17	.539^#^
10,11‐EDP	0.01 ± 0.01	0.01 ± 0.01	.222^#^
13,14‐EDP	n.d.	n.d.	n/a
16,17‐EDP	n.d.	n.d.	n/a
19,20‐EDP	0.06 ± 0.05	0.11 ± 0.22	.505^#^
7,8‐DiHDPA	n.d.	n.d.	n/a
10,11‐DiHDPA	n.d.	n.d.	n/a
13,14‐DiHDPA	n.d.	n.d.	n/a
16,17‐DiHDPA	0.01 ± 0.01	0.02 ± 0.01	.461^#^
19,20‐DiHDPA	0.12 ± 0.06	0.15 ± 0.14	.744^#^
LOX/CYP ω/(ω−1) metabolites			
13‐HODE	**8.96 ± 4.64**	**36.76 ± 31.23**	**<.001^#^**
5‐HETE	**0.21 ± 0.07**	**0.60 ± 0.37**	**<.001^#^**
8‐HETE	**0.28 ± 0.14**	**0.90 ± 0.59**	**<.001^#^**
9‐HETE	**0.55 ± 0.32**	**1.85 ± 1.46**	**<.001^#^**
11‐HETE	**0.84 ± 0.32**	**2.66 ± 1.64**	**<.001^#^**
12‐HETE	**4.23 ± 2.53**	**28.11 ± 33.78**	**<.001^#^**
15‐HETE	**0.65 ± 0.25**	**2.15 ± 1.05**	**<.001**
16‐HETE	**0.10 ± 0.03**	**0.15 ± 0.06**	**.003**
17‐HETE	n.d.	n.d.	n/a
18‐HETE	n.d.	n.d.	n/a
19‐HETE	n.d.	n.d.	n/a
20‐HETE	0.10 ± 0.05	0.10 ± 0.04	.877
12‐HpETE	n.d.	n.d.	n/a
5‐HEPE	**0.03 ± 0.02**	**0.14 ± 0.34**	**.021^#^**
8‐HEPE	**0.04 ± 0.03**	**0.32 ± 0.87**	**<.001^#^**
9‐HEPE	**0.05 ± 0.04**	**0.35 ± 0.96**	**.003^#^**
12‐HEPE	**0.97 ± 0.52**	**8.06 ± 14.72**	**.006^#^**
15‐HEPE	**0.06 ± 0.04**	**0.70 ± 1.82**	**<.001^#^**
18‐HEPE	**0.12 ± 0.06**	**1.52 ± 3.96**	**<.001^#^**
19‐HEPE	0.03 ± 0.02	0.22 ± 0.69	.367^#^
20‐HEPE	n.d.	n.d.	n/a
4‐HDHA	**0.03 ± 0.02**	**0.18 ± 0.32**	**.001^#^**
7‐HDHA	**0.02 ± 0.01**	**0.11 ± 0.05**	**.001^#^**
8‐HDHA	**0.04 ± 0.02**	**0.22 ± 0.35**	**<.001^#^**
10‐HDHA	**0.06 ± 0.03**	**0.63 ± 1.07**	**<.001^#^**
11‐HDHA	**0.19 ± 0.08**	**0.87 ± 1.32**	**<.001^#^**
13‐HDHA	**0.08 ± 0.04**	**0.44 ± 0.61**	**<.001^#^**
14‐HDHA	**0.35 ± 0.17**	**2.81 ± 3.60**	**<.001^#^**
16‐HDHA	**0.07 ± 0.03**	**0.37 ± 0.63**	**<.001^#^**
17‐HDHA	**0.42 ± 0.15**	**2.59 ± 4.22**	**<.001^#^**
20‐HDHA	0.27 ± 0.09	0.67 ± 1.00	.050^#^
21‐HDHA	**0.11 ± 0.05**	**0.42 ± 0.59**	**.002^#^**
22‐HDHA	**0.72 ± 0.29**	**1.27 ± 0.71**	**.013**

Note

Bold font indicates statistical significance.

Abbreviations: n.d., not detected; n/a, not applicable.

**Table 2:** Comparison of epoxy‐metabolites and their ratios between control subjects versus CKD patients before hemodialysis (HD) (*n* = 15 each)

(A) Concentrations of individual total epoxides together or their respective diols in RBCs			
Epoxides or Diols (ng/g)	Control (Mean ± *SD*)	HD (Mean ± *SD*)	*p*‐value, Mann‐Whitney test
9,10‐EpOME+12,13‐EpOME	43.03 ± 21.07	36.10 ± 10.60	.3195
9,10‐DiHOME+12,13‐DiHOME	6.377 ± 2.104	8.049 ± 3.178	.0971
5,6‐EET+8,9‐EET+11,12 EET+14,15‐EET	315.3 ± 51.27	285.9 ± 86.25	.2998
5,6‐DHET+8,9‐DHET+11,12‐DHET+14,15‐DHET	**2.986 ± 0.5208**	**4.477 ± 2.789**	**.0421**
5,6‐EEQ+8,9‐EEQ+11,12‐EEQ+14,15‐EEQ+17,18‐EEQ	**50.81 ± 16.35**	**63.65 ± 120.5**	**.0225**
5,6‐DiHETE+8,9‐DiHETE+11,12‐DiHETE+14,15‐DiHETE+17,18‐DiHETE	0.2153 ± 0.1021	0.3420 ± 0.7263	.1835
7,8‐EDP+10,11‐EDP+13,14‐EDP+16,17‐EDP+19,20‐EDP	28.40 ± 9.805	28.86 ± 14.26	.6187
7,8‐DiHDPA+10,11‐DiHDPA+13,14‐DiHDPA+16,17‐DiHDPA+19,20‐DiHDPA	**0.6813 ± 0.2123**	**1.039 ± 0.5678**	**.0464**

(B) Ratios estimated using total concentrations of epoxides and diols in RBCs			
Ratios	Control (Mean ± *SD*)	HD (Mean ± *SD*)	*p*‐value, Mann‐Whitney test
Ratio (9,10‐DiHOME+12,13‐DiHOME)/(9,10‐EpOME+12,13‐EpOME)	0.1628 ± 0.06583	0.2425 ± 0.1255	.0564
Ratio (5,6‐DHET+8,9‐DHET+11,12‐DHET+14,15‐DHET)/(5,6‐EET+8,9‐EET+11,12 EET+14,15‐EET)	**0.0096 ± 0.001705**	**0.01652 ± 0.009067**	**.0279**
Ratio (5,6‐DiHETE+8,9‐DiHETE+11,12‐DiHETE+14,15‐DiHETE+17,18‐DiHETE)/(5,6‐EEQ+8,9‐EEQ+11,12‐EEQ+14,15‐EEQ+17,18‐EEQ)	0.00416 ± 0.001188	0.005927 ± 0.004070	.2627
Ratio (7,8‐DiHDPA+10,11‐DiHDPA+13,14‐DiHDPA+16,17‐DiHDPA+19,20‐DiHDPA)/(7,8‐EDP+10,11‐EDP+13,14‐EDP+16,17‐EDP+19,20‐EDP)	**0.02445 ± 0.005347**	**0.03765 ± 0.01382**	**.0025**

Note

Bold font indicates statistical significance.

### 3.3 Ratios

The main route of EpOMEs, EETs, EEQs, and EDPs metabolism in many cells is conversion into DiHOMEs, DHETs, dihydroxyeicosatetraenoic acids (DiHETEs), and dihydroxydocosapentaenoic acids (DiHDPAs) by the sEH, respectively (Figure 1). To provide possible insights into the nature of the observed accumulation of DHETs, EEQs, and DiHDPAs in RBCs of ESRD patients, we calculated diol/epoxide ratios in RBCs and compared the results with the control subjects (Table 2B). We found that the four classes of epoxy‐metabolites are unequally hydrolyzed and appear in the RBCs (Table 2B for controls). Compared to EETs and EEQs (ratios diols/epoxy‐metabolites, 0.0096 ± 0.0017 vs. 0.0042 ± 0.00012, Dunn's multiple comparison test, *p* > .05), EpOMEs and EDPs (ratios diols/epoxy‐metabolites, 0.1628 ± 0.0658 vs. 0.0244 ± 0.0053, Dunn's multiple comparison test, *p* > .05) are preferentially metabolized into their diols. In fact, the following order of ratios was identified: DiHOMEs/EpOMEs=DiHDPA/EDPs>DHETs/EETs=DiHETEs/EEQs (Dunn's multiple comparison test, *p* < .05). ESRD patients showed increased ratios for DHET/EET and DiHDPA/EDP, which indicates that increased sEH activity preferred for EET and EDP substrate classes in vivo may have caused the observed accumulation of 8,9‐DHET, 14,15‐DHET, 7,8‐DiHDPA, 10,11‐DiHDPA, 13,14‐DiHDPA, and 16,17‐DiHDPA in the RBCs in ESRD. The observed accumulation of EEQs is unlikely to result from changes in sEH activity (Table 2B) or accumulation of eicosapentaenoic acid (EPA) as EPA levels are not increased in RBCs of our patients (Gollasch et al., 2020) (Figure 1).

### 3.4 Effects of hemodialysis

With the exception of 7,8‐DiHDPA, the data (Table 3) demonstrate no change of total CYP epoxides and LOX/CYP ω/(ω‐1)‐hydroxylase metabolites in response to a single dialysis (Table 3A). Accordingly, the diol/epoxide ratios were not altered (Table 4). However, hemodialysis treatment increased several CYP epoxides and LOX/CYP ω/(ω‐1)‐hydroxylase metabolites in free state, such as 11,12‐DHET, 13‐HODE, 5‐HETE, 8‐HETE, 9‐HETE, 11‐HETE, 15‐HETE, 5‐HEPE, 8‐HDHA, 10‐HDHA, 13‐HDHA, 16‐HDHA, and 17‐HDHA (Table 3B).

**Table 3:** Effects of hemodialysis on epoxy‐ and hydroxy‐metabolites in the CKD patients before (pre‐HD) and at cessation (post‐HD) of hemodialysis (*n* = 15 each)

Amount, (ng/g)	Pre‐HD (Mean ± *SD*)	Post‐HD (mean ± *SD*)	*p* value, paired *t* test (^#^paired Wilcoxon test)
(A) Total metabolites in RBCs			
CYP epoxy‐metabolites			
(a) EpOMEs/DiHOMES			
9,10‐EpOME	25.48 ± 6.59	25.91 ± 5.94	.802
12,13‐EpOME	10.62 ± 6.16	11.52 ± 7.93	.307^#^
9,10‐DiHOME	5.13 ± 1.92	5.27 ± 1.42	.623
12,13‐DiHOME	2.92 ± 1.45	3.00 ± 0.90	.914
(b) EETs/DiHOMEs			
5,6‐EET	148.54 ± 44.94	162.71 ± 46.95	.198
8,9‐EET	39.90 ± 9.00	43.76 ± 8.50	.134
11,12‐EET	37.86 ± 11.98	41.54 ± 11.54	.112
14,15‐EET	59.58 ± 22.69	63.97 ± 21.75	.162
5,6‐DHET	0.98 ± 0.43	1.06 ± 0.43	.117
8,9‐DHET	2.03 ± 1.81	2.13 ± 1.67	.112^#^
11,12‐DHET	0.96 ± 0.61	0.99 ± 0.50	.334^#^
14,15‐DHET	0.51 ± 0.16	0.53 ± 0.12	.148
(c) EEQs/DiHETEs			
8,9‐EEQ	3.51 ± 6.41	3.39 ± 5.75	1.000^#^
5,6‐EEQ	51.78 ± 98.53	45.89 ± 69.79	.650^#^
11,12‐EEQ	2.56 ± 4.74	2.40 ± 3.49	.125^#^
14,15‐EEQ	1.91 ± 3.5	1.66 ± 2.55	.910^#^
17,18‐EEQ	3.90 ± 7.31	3.66 ± 5.81	.460^#^
5,6‐DiHETE	0.28 ± 0.49	0.24 ± 0.32	.733^#^
8,9‐DiHETE	n.d.	n.d.	n/a
11,12‐DiHETE	n.d.	n.d.	n/a
14,15‐DiHETE	n.d.	n.d.	n/a
17,18‐DiHETE	n.d.	n.d.	n/a
(d) EDPs/DiHDPAs			
7,8‐EDP	18.16 ± 12.19	19.48 ± 12.59	.307^#^
10,11‐EDP	1.35 ± 0.47	1.50 ± 0.73	.427^#^
13,14‐EDP	0.44 ± 0.15	0.52 ± 0.31	.551^#^
16,17‐EDP	4.72 ± 1.78	5.46 ± 2.50	.078^#^
19,20‐EDP	4.22 ± 1.52	5.14 ± 2.84	.109
7,8‐DiHDPA	**0.40 ± 0.30**	**0.48 ± 0.42**	**.036^#^**
10,11‐DiHDPA	0.09 ± 0.05	0.10 ± 0.07	.256^#^
13,14‐DiHDPA	0.11 ± 0.04	0.12 ± 0.04	.363^#^
16,17‐DiHDPA	0.19 ± 0.06	0.20 ± 0.07	.124
19,20‐DiHDPA	0.26 ± 0.16	0.27 ± 0.14	.173^#^
LOX/CYP ω/(ω−1) metabolites			
13‐HODE	77.47 ± 18,89	82.00 ± 18.35	.391
5‐HETE	53.45 ± 14.83	56.62 ± 10.08	.295
8‐HETE	35.11 ± 10.20	36.63 ± 7.23	.379
9‐HETE	37.84 ± 9.77	39.89 ± 7.07	.268
11‐HETE	54.16 ± 14.84	56.92 ± 10.96	.323
12‐HETE	43.47 ± 12.68	45.33 ± 8.36	.466
15‐HETE	93.95 ± 24.59	99.31 ± 18.34	.281
16‐HETE	4.91 ± 1.43	5.14 ± 1.08	.412
17‐HETE	0.22 ± 0.10	0.22 ± 0.08	.363^#^
18‐HETE	0.32 ± 0.21	0.34 ± 0.23	.112^#^
19‐HETE	0.42 ± 0.11	0.49 ± 0.17	.085
20‐HETE	0.62 ± 0.08	0.65 ± 0.23	.602
12‐HpETE	n.d.	n.d.	
5‐HEPE	2.05 ± 2.64	2.30 ± 3.25	.281^#^
8‐HEPE	1.15 ± 1.55	1.26 ± 1.88	.363^#^
9‐HEPE	1.35 ± 1.64	1.51 ± 2.11	.281^#^
12‐HEPE	2.15 ± 3.12	2.29 ± 3.38	.307^#^
15‐HEPE	2.06 ± 2.74	2.23 ± 2.95	.053^#^
18‐HEPE	5.28 ± 7.10	5.63 ± 7.64	.140^#^
19‐HEPE	1.89 ± 2.80	1.80 ± 2.32	.910^#^
20‐HEPE	n.d.	n.d.	
4‐HDHA	11.20 ± 4.61	12.71 ± 5.81	.140^#^
7‐HDHA	5.90 ± 2.69	6.33 ± 2.85	.233^#^
8‐HDHA	7.16 ± 3.11	7.74 ± 3.35	.112^#^
10‐HDHA	8.05 ± 3.79	8.57 ± 3.93	.334^#^
11‐HDHA	9.43 ± 4.47	10.03 ± 4.90	.140^#^
13‐HDHA	10.43 ± 4.20	11.22 ± 4.95	.173^#^
14‐HDHA	6.82 ± 3.38	7.41 ± 3.41	.156^#^
16‐HDHA	9.80 ± 3.88	10.55 ± 4.14	.112^#^
17‐HDHA	15.55 ± 6.92	16.83 ± 7.47	.078^#^
20‐HDHA	22.57 ± 9.88	24.53 ± 10.60	.112^#^
21‐HDHA	3.76 ± 1.70	3.71 ± 1.32	.790
22‐HDHA	n.d.	n.d.	n/a
(B) Free metabolites in RBCs			
CYP epoxy‐metabolites			
(a) EpOMEs/DiHOMES			
9,10‐EpOME	1.79 ± 1.00	2.08 ± 0.48	.156^#^
12,13‐EpOME	1.25 ± 0.91	1.89 ± 0.88	.053^#^
9,10‐DiHOME	0.52 ± 0.34	0.65 ± 0.29	.147
12,13‐DiHOME	2.20 ± 1.52	2.91 ± 1.87	.256^#^
(b) EETs/DiHOMEs			
5,6‐EET	0.45 ± 0.19	0.54 ± 0.21	.114
8,9‐EET	0.06 ± 0.04	0.07 ± 0.10	.480^#^
11,12‐EET	0.20 ± 0.08	0.21 ± 0.06	.654
14,15‐EET	0.74 ± 0.36	0.93 ± 0.35	.100^#^
5,6‐DHET	<0.01 ± 0.01	<0.01 ± 0.01	n/a
8,9‐DHET	0.02 ± 0.01	0.03 ± 0.03	.131^#^
11,12‐DHET	**<0.02 ± 0.01**	**0.02 ± 0.01**	**.005**
14,15‐DHET	0.01 ± 0.01	0.02 ± 0.01	.427^#^
(c) EEQs/DiHETEs			
5,6‐EEQ	0.90 ± 3.39	1.08 ± 3.52	.068^#^
8,9‐EEQ	0.31 ± 0.50	0.20 ± 0.55	.128^#^
11,12‐EEQ	0.07 ± 0.14	0.07 ± 0.09	.424^#^
14,15‐EEQ	0.20 ± 0.23	0.17 ± 0.24	.477^#^
17,18‐EEQ	0.53 ± 1.02	0.49 ± 0.80	.955^#^
5,6‐DiHETE	<0.01 ± 0.01	<0.01 ± 0.01	.477
8,9‐DiHETE	<0.01	<0.01	n/a
11,12‐DiHETE	<0.01	<0.01	n/a
14,15‐DiHETE	0.01 ± 0.04	0.02 ± 0.04	.394^#^
17,18‐DiHETE	0.11 ± 0.23	0.16 ± 0.38	.394^#^
(d) EDPs/DiHDPAs			
7,8‐EDP	0.17 ± 0.17	0.22 ± 0.18	.112^#^
10,11‐EDP	0.01 ± 0.01	0.01 ± 0.01	.463^#^
13,14‐EDP	n.d.	n.d.	n/a
16,17‐EDP	n.d.	n.d.	n/a
19,20‐EDP	0.11 ± 0.22	0.09 + 0.09	.507^#^
7,8‐DiHDPA	n.d.	n.d.	n/a
10,11‐DiHDPA	<0.01 ± 0.01	<0.01 ± 0.01	n/a
13,14‐DiHDPA	<0.01 ± 0.01	0.01 ± 0.01	.465^#^
16,17‐DiHDPA	0.02 ± 0.01	0.03 ± 0.02	.140^#^
19,20‐DiHDPA	0.15 ± 0.14	0.18 ± 0.18	.334^#^
LOX/CYP ω/(ω−1) metabolites			
13‐HODE	**36.76 ± 31.23**	**45.70 ± 31.56**	**.031^#^**
5‐HETE	**0.60 ± 0.37**	**0.85 ± 0.53**	**.023^#^**
8‐HETE	**0.90 ± 0.59**	**1.24 ± 0.83**	**.008^#^**
9‐HETE	**1.85 ± 1.46**	**2.51 ± 1.84**	**.031^#^**
11‐HETE	**2.66 ± 1.64**	**3.37 ± 2.16**	**.017^#^**
12‐HETE	28.11 ± 33.78	34.20 ± 33.78	.334^#^
15‐HETE	**2.15 ± 1.05**	**2.78 ± 1.54**	**.008^#^**
16‐HETE	0.15 ± 0.06	0.15 ± 0.04	.999
17‐HETE	n.d.	n.d.	n/a
18‐HETE	n.d.	n.d.	n/a
19‐HETE	n.d.	n.d.	n/a
20‐HETE	0.10 + 0.04	0.12 ± 0.06	.155
12‐HpETE	n.d.	n.d.	n/a
5‐HEPE	**0.14 ± 0.34**	**0.18 ± 0.45**	**.031^#^**
8‐HEPE	0.32 ± 0.87	0.35 ± 0.98	.394^#^
9‐HEPE	0.35 ± 0.96	0.42 ± 1.14	.112^#^
12‐HEPE	8.06 ± 14.72	10.61 ± 21.49	.191^#^
15‐HEPE	0.70 ± 1.82	0.75 ± 1.96	.307^#^
18‐HEPE	1.52 ± 3.96	1.53 ± 3.96	.776^#^
19‐HEPE	0.22 ± 0.69	0.21 ± 0.64	.955^#^
20‐HEPE	n.d.	n.d.	n/a
4‐HDHA	0.18 ± 0.32	0.25 ± 0.45	.061^#^
7‐HDHA	0.11 ± 0.05	0.15 ± 0.28	.112^#^
8‐HDHA	**0.22 ± 0.35**	**0.31 ± 0.49**	**.031^#^**
10‐HDHA	**0.63 ± 1.07**	**0.79 ± 1.39**	**.023^#^**
11‐HDHA	0.87 ± 1.32	1.07 ± 1.60	.100^#^
13‐HDHA	**0.44 ± 0.61**	**0.55 ± 0.74**	**.036^#^**
14‐HDHA	2.81 ± 3.60	3.40 ± 4.40	.078^#^
16‐HDHA	**0.37 ± 0.63**	**0.50 ± 0.93**	**.012^#^**
17‐HDHA	**2.59 ± 4.22**	**3.35 ± 5.24**	**.031^#^**
20‐HDHA	0.67 ± 1.00	0.83 ± 1.37	.112^#^
21‐HDHA	0.42 ± 0.59	0.48 ± 0.68	.256^#^
22‐HDHA	1.27 ± 0.71	1.30 ± 0.78	.837

Note

Bold font indicates statistical significance.

**Table 4:** Effects of hemodialysis on epoxide and their respective diol ratios in the CKD patients before (pre‐HD) and at cessation (post‐HD) of hemodialysis (*n* = 15 each). Ratios were estimated using total concentrations of epoxides and diols in RBCs

Ratios	Pre‐HD (Mean ± *SD*)	Post‐HD (Mean ± *SD*)	*p*‐value, Paired Wilcoxon test)
Ratio (9,10‐DiHOME+12,13‐DiHOME)/(9,10‐EpOME+12,13‐EpOME)	0.2425 ± 0.1255	0.2435 ± 0.1043	.8904
Ratio (5,6‐DHET+8,9‐DHET+11,12‐DHET+14,15‐DHET)/(5,6‐EET+8,9‐EET+11,12 EET+14,15‐EET)	0.01652 ± 0.009067	0.01623 ± 0.008816	.8647
Ratio (5,6‐DiHETE+8,9‐DiHETE+14,15‐DiHETE+17,18‐DiHETE)/(5,6‐EEQ+8,9‐EEQ+11,12‐EEQ+14,15‐EEQ+17,18‐EEQ)	0.005927 ± 0.004070	0.005647 ± 0.003565	.4896
Ratio (7,8‐DiHDPA+10,11‐DiHDPA+13,14‐DiHDPA+16,17‐DiHDPA+19,20‐DiHDPA)/(7,8‐EDP+10,11‐EDP+13,14‐EDP+16,17‐EDP+19,20‐EDP)	0.03765 ± 0.01382	0.03873 ± 0.01658	.4887

## 4 DISCUSSION

Our data demonstrate that RBCs of ESRD patients accumulated three CYP epoxide classes (DHETs, EEQs, and DiHDPAs) and various HETEs, including 5‐HETE, 8‐HETE, 9‐HETE, 11‐HETE, 12‐HETE, 15‐HETE, and 19‐HETE, compared to control subjects. Furthermore, hemodialysis treatment is insufficient to change the total concentrations of these and other LOX/CYP metabolites in RBCs of ESRD patients. Since the four subclasses of CYP epoxy metabolites increase in plasma after the dialysis treatment (Gollasch et al., 2020), we suggest that total CYP metabolites in RBCs are relatively invulnerable in CKD and hemodialysis (possibly due to slow exchange). Of note, ESRD is associated with increased levels of several free CYP epoxides and LOX/CYP ω/(ω‐1)‐hydroxylase metabolites in RBCs. Since several of those mediators are also increased by hemodialysis treatment itself, we suggest that free RBC eicosanoids constitute a fraction of lipid mediators, which are particularly vulnerable in CKD and hemodialysis. The extent to which the RBC eicosanoids exhibit beneficial or detrimental cardiovascular effects in CKD, possibly in comprehensive lipidomic (patho)physiological networks, remains to be explored. Nonetheless, our results indicate that RBCs could represent a reservoir for PUFA CYP epoxy‐metabolites and LOX/CYP hydroxy metabolites, which on release may act in a vasoregulatory capacity to affect cardiovascular responses in hemodialysis patients.

### 4.1 EETs

RBCs are reservoir of EETs which on release may act in a vasoregulatory capacity (Jiang et al., 2010, 2011). In addition to serving as carriers of O_2_, RBCs are known to regulate the microvascular perfusion by liberating adenosine triphosphate (ATP) and EETs upon exposure to a low O_2_ environment (Jiang et al., 2010; Sprague et al., 2010). The release of EETs is activated by P2X_7_ receptor stimulation *via* ATP to cause the circulatory response (Jiang et al., 2007). RBCs are believed to serve as a source of plasma EETs, which are esterified to the phospholipids of lipoproteins. Therefore, levels of free EETs in plasma are found to be low (~3% of circulating EETs) (Jiang et al., 2010, 2011). Erythro‐EETs are produced by direct oxidation of AA and the monooxygenase‐like activity of hemoglobin (Jiang et al., 2010, 2011, 2012). On release, EETs and their diols (DHETs) produce vasodilation (Hercule et al., 2009; Lu et al., 2001), are pro‐fibrinolytic and reduce inflammation (Jiang et al., 2010, 2011, 2012). Exhaustive exercise increases the circulating levels of 5,6‐DHET (Gollasch et al., 2019). In this study, we were able to demonstrate that RBCs of ESRD patients show increased accumulation of total DHETs. In particular, we observed increases in total concentrations of 8,9‐DHET and 14,15‐DHET in the RBCs. Hemodialysis did not affect this accumulation. It remains unknown whether RBCs are capable of liberating erythro‐DHETs into the blood and/or tissues in kidney patients. Our results indicate that CKD affects the RBC reservoir for DHETs, but not EETs, which on release may affect the cardiovascular response.

### 4.2 Other PUFA metabolites

We observed increases in total concentrations of EEQs (5,6‐EEQ, 11,12‐EEQ, 14,15‐EEQ, 17,18‐EEQ) and EDP/DiHDPAs (19,20‐EDP, 7,8‐DiHDPA, 10,11‐ DiHDPA, 13,14‐DiHDPA, 16,17‐DiHDPA) and HETEs (5‐HETE, 8‐HETE, 9‐HETE, 11‐HETE, 12‐HETE, 15‐HETE, 19‐HETE) in RBCs of our ESRD patients. Little is known about the functions of EEQs and EDPs. Both EEQs and EDPs are potent vasodilators (Hercule et al., 2007; Lauterbach et al., 2002; Morin et al., 2011; Ulu et al., 2014). EDPs have antiangiogenic (McDougle et al., 2017), anti‐fibrotic (Sharma et al., 2016) and protective effects in post‐ischemic functional recovery, at least in particular by maintaining mitochondrial function and reducing inflammatory responses (Arnold et al., 2010; Darwesh et al., 2019). It is possible that their diols (DiHDPAs) are also biologically active and may exert beneficial effects in cardiac arrhythmias (Zhang et al., 2016). DiHDPAs dilate coronary microvessels with similar potency to EEQ isomers in canine and porcine models (Zhang et al., 2001) and inhibit human platelet aggregation with moderately lower potency to EDPs and EEQs (VanRollins, 1995). Specific 17,18‐EEQ analogs are in development to serve as novel antiarrhythmic agents (Adebesin et al., 2019). HETEs are involved in many chronic diseases such as inflammation, obesity, cardiovascular disease, kidney disease, and cancer, for review see (Gabbs et al., 2015). Nonetheless, it remains unknown whether RBCs are capable of liberating EEQs, DiHDPAs, or HETEs into blood or tissues. Our data indicate that both metabolite classes are novel candidates potentially released by RBCs to exhibit cardiovascular effects in health and CKD.

Surprisingly, we did detect increases in various free CYP epoxides and LOX/CYP ω/(ω‐1)‐hydroxylase metabolites in RBCs in ESRD, which were augmented by hemodialysis. The mechanism by which CKD and hemodialysis raises the levels of those erythro‐metabolites is not known. Since those metabolites cannot be synthesized endogenously in appreciable amounts, accelerated release into and uptake from plasma could be a possible explanation. The more pronounced changes observed in free metabolite levels within the RBCs, as compared with the total RBC compartment, indicate that free erythro‐eicosanoids should be considered more dynamic or vulnerable with respect to metabolite flux. The design of our study does not differentiate between patient groups undergoing long‐term dialysis therapy with regard to the specific underlying renal disease. Nevertheless, the impact of those epoxides and hydroxy metabolites has yet to be integrated into a (patho)physiological context.

## 5 CONCLUSIONS

Our results show that CKD affects the levels of numerous CYP epoxides and hydroxy metabolites (DHETs, EEQs, DiHDPAs, and HETEs) in circulating RBCs compared to control subjects, which on release may act in a vasoregulatory capacity. Although hemodialysis treatment was insufficient to change the majority of those total metabolites, we detected pronounced changes in free metabolite levels within the ESRD RBCs and in response to hemodialysis, indicating that free erythro‐epoxides could also contribute to the cardiovascular risk, for example, in diabetes or hypertension. More research is needed to determine the contribution of RBC epoxy‐ and hydroxy‐metabolites to cardiac performance and blood pressure regulation in health, cardiovascular, and specific kidney diseases.

## CONFLICT OF INTEREST

None.

## AUTHOR CONTRIBUTIONS

BG, MG, and FCL planned and designed the experimental studies. MR and ID performed the LC–MS/MS spectrometry experiments. All authors contributed to the implementation and analyses of the experiments. BG drafted the article, and all authors, contributed to its completion.
